# Case of Necrosis of the Tibia of the Right Leg

**Published:** 1805-10-01

**Authors:** Eloy Berger

**Affiliations:** New-York


### CASE *of* NECROSIS *of the* TIBIA *of the* RIGHT LEG: *by* *Dr.* ELOY BERGER, *of New-York.*

[ Extracted from the New-York Medical Repository ]

IN the month of April, 1803, I was called upon, in the absence of M. Delmas, my colleague, by Mr. R. Kemble, to attend one of his servants, who had been laid up with a sore leg nine or ten months, and had used various kinds of treatment and application without any success. Mr. Kemble, feeling very much for the unfortunate man, requested me to inspect his leg with great attention, that I might give him my opinion about the nature of his complaint. Upon the first inspection of the leg, I found it considerably swollen, from the tuberosity of the tibia, down to three inches above the articulation of the ancle; the swelling affected the anterior part of the leg; it was very hard, and seemed to be formed by a bony substance; several ulcerations were spread over its surface. At the first sight I suspected it to be a Necrosis; but to ascertain it, I examined, with a small probe, all the ulcerated part, and, and, upon due inquiry, I found divers fistulas, into which I introduced the probe to discover their depth in the bone. One of them, which was a little below the tuberosity of the tibia, was more than an inch deep, and two or three others in the same direction below that, had about the same depth. By this examination, I was convinced that every fistula had a communication with the centre of the bony cylinder. The interval between the upper and the lower fistula was about four inches. From these inquiries, I entertained no uncertainty about the nature of the complaint: I assured Mr. K. that his servant had a necrosis, which could not be cured but by a chirurgical operation. He immediately wished to have him committed to my care and that of M. Delmas, who had already been very useful to the patient on occasions which had preceded the formation of this complaint. On my return from Mr. K's country seat, I communicated to M. Delmas my opinion concerning the nature of the disease, assuring him that I was certain that a piece of bone was separated in the centre of the bony mass supporting the leg, and which could not be extracted but by the removal of a sufficient portion of the bone forming the case in which it was confined. I consequently proposed to him the operation, which I had before performed several times. He agreed with me, and a few days after we performed it.

To have the patient more at hand, he came into New-York, to Mr. Peter Kemble's, where, after the usual preparation, we performed the operation in the following manner. We made at first, two half-eliptic incisions, in which we included all the fistulas. These incisions were begun a little below the tuberosity of the tibia, and ended together about half an inch lower than the last fistula. We held, with a small forceps, the upper extremity of the incised skin, and dissected it downwards in its totality. By that process, the bone was laid bare five inches in length, and three in breadth. After having washed away, with a spunge, the blood which covered its surface, we perceived it not to be even as the tibia is commonly, but uneven and rough. We then cut off, with a gouge and a mallet, many pieces of the bony substance, that we might reach the middle of the cylinder, in which we supposed the disease to exist. With much patience and courage we attained it, and forthwith we discovered a piece of bone, much whiter than that which we had been obliged to cut off, in order to discover it. Having taken hold of it with the forceps, forceps, we perceived it to be loose, and that it was only necessary to lay it entirely bare to extract it easily. We then persisted in cutting off with the gouge and mallet, the whole thickness of the bony mass which kept it confined, down to the lower fistula; and notwithstanding our being retarded in the operation by the discharge of blood, which we were constantly obliged to wash away with the spunge, we laid bare in twenty minutes the whole of the detached piece of bone. Its extraction was then the easier, as it had no other adhesion to the sound parts but by the crossing of the angles of each other. After this operation, we washed the blood out of the bony cavity, and cleared away the matter with which it was filled. We made round the sharp angles which were in it, we filled it with dry lint, and secured the whole by compresses and a bandage.

Afterwards we examined this piece of bone; it was four inches in length, and contained in its upper extremity the three substances of the bones; it was white and smooth, and much harder than the pieces which we had cut off, in order to reach it.

One might be surprised at the patient having not, during all the time of the operation, which lasted more than twenty minutes, uttered a single expression of pain; we even observed that he made no complaint but during the incision of the skin. Four days after the operation, the suppuration of the wound began to be established, without the patient having had any fever: he had taken nothing during that time but some lemonade for his drink, and some soup for his food. At first, I dressed the wound with small dossils, imbued with vulnerary water, which was employed to promote the filling up of its cavity; I covered them with a pledgit of lint, over which I laid a poultice of bread and water, and kept the whole secure by a bandage. The same dressing was continued for a long time without any alteration. Hardly were ten days elapsed before I perceived many fleshy granulations, arising all over the bony surface, which would have soon filled its cavity, had I not opposed their increasing, by the interposition of the small dosils, and a slight compression made upon the wound: this restriction kept down fungous flesh, and seemed necessary, in order to keep the sore in a state of firmness and moderate inflammation, which is necessary for a good suppuration. In this manner, the pus, during all the time of my attendance, was white, entirely inodorous, and the the ulcer drawing very fast to a cicatrization. The dressing was constantly the same, except that I laid aside the vulnerary water to use only dry lint, and lessened the dossils in proportion to the diminution of the cavity of the ulcer. In short, four months after the operation, it was perfectly healed.

From my inquiries to discover the cause of this disease, I was informed that the patient had been affected some time before with an inflammatory rheumatism; that the humour having fixed upon the right leg, a considerable swelling ensued, and that several abscesses had, after very long sufferings, been the result of it. Such is the information I was able to obtain about the cause of this complaint.

### *Reflections on this Case.*

From the information just mentioned, and the nature of the complaint, is it not natural to think, that one of the abscesses which affected the leg, took its seat between the periosteum and the bone? that the pus formed between those two parts, necessarily destroyed the vessels of communication between them? that those vessels which are destined to convey into the bone the materials of nutrition being destroyed, those materials, instead of reaching the place of their destination, remained in the periosteum, spread in the different meshes with which it is framed, and formed a bony production, the configuration of which was necessarily that of the membrane which formed its mould? But, what could have become of the part of the bone laying under it? Receiving no longer the aliment of its reparation, it must have perished and fallen into mortification—certainly it must; and it is on that account, that provident Nature had begun the bony production to repair the loss she was sustaining: the one seems even to have been formed, whilst the other was falling into ruin. Whoever examines attentively this disease, will acknowledge that course of Nature in its formation. But it is obvious, that it is not sufficient for her to have thus repaired the loss she sustains; but she must, besides, get rid of the piece of the bone fallen into mortification, and that part of her task is not the easiest to perform; it is in fact the more difficult, as the piece of bone is confined in a kind of case; yet the difficulty does not stop her. That wise and powerful mother finds remedies even in the most difficult cases, and if she do not succeed always in fulfilling her design, an attentive observer perceives, at least, that she she neglects nothing to accomplish it. This is the way which she employs in that occurrence to get rid of the detached body, whose presence is troublesome to her. Wise in all her operations, that body is the agent she makes use of to obtain a vehicle proper to convey it out; it is, in fact, its presence, which, by irritating the internal part of the case in which it is confined, determines in it a more or less abundant suppuration; the matter produced soon makes several holes through the bony case, and then pierces the skin to find vent. But as the pus, before its evacuation, lies in contact, during a certain time, with the detached piece of bone, it separates, by a kind of maceration, some small particles of it, which it conveys continually out, and would succeed, in time, in carrying off the whole of the disease, if circumstances were not unfavourable.

But it is easy to perceive that these efforts, however wonderful, must often prove insufficient to procure a perfect recovery, when the detached piece of bone is considerable. Nature is too slow in her operations, and the patient often falls away before the accomplishment of her design: then it is the part of our art to make up for the weakness of Nature, and to furnish the means she wants; she has traced out what must be done; she has fulfilled her task—the surgeon must fulfil his. Armed with a gouge and a mallet, he must then cut off, as we have done in the case above, a part of the bony cylinder which conceals the disease, in order to be able to extract it easily. In that manner our art performs, in a few minutes, what Nature could not do in several years; it prevents many severe occurrences, which always attend, in process of time, this distressing disease, and which, if they do not bring the patient to the grave, at least necessitate an amputation, always painful, and often fatal. We can assert, that four patients, upon whom we have practised this operation, have suffered no unfavourable consequences in the warm climates, where tetanic affections very often follow the great operations.

FATAL

